# EFFECT OF PARTICIPATORY ERGONOMICS PROGRAM ON REDUCING ERGONOMIC RISK FACTORS AMONG THE AGING WORKERS ON GINGER PEELERS IN WAT PURANAWAS COMMUNITY, THAILAND

**DOI:** 10.13075/ijomeh.1896.02486

**Published:** 2025

**Authors:** Surachart Thongchoomsin, Supatida Sorasak Siangchin, Anuchart Kaunnil, Phichaya Baramee

**Affiliations:** 1 Mahidol University, Occupational Therapy Division, Faculty of Physical Therapy, Nakhon Pathom, Thailand; 2 Chiang Mai University, Department of Occupational Therapy, Faculty of Associated Medical Sciences, Chiang Mai, Thailand

**Keywords:** aging, ergonomics, work performance, occupational health, musculoskeletal disorders, community-based participatory research

## Abstract

**Objectives:**

The present study aims to investigate the effects of the participatory ergonomics (PE) program on reducing the ergonomic risk factors and increasing the work performance among the aging workers who work as ginger peelers in the Wat Puranawas community in Thailand.

**Material and Methods:**

Fourteen older workers who work in the Wat Puranawas community are recruited, by purposive sampling. Participants attend the PE program twice, each session lasts 60 min. The measuring instruments used are: the *Rapid Upper Limb Assessment* (RULA), the *Work Ability Index* (WAI), and the *Canadian Occupational Performance Measure* (COPM). The Friedman test is used for comparing the difference in the outcome from 3 periods, the pre-test period, the post-test period (at 2 weeks after implementing the PE program), and the follow-up period (at 4 weeks after the conclusion of the PE program). Wilcoxon signed-rank test was also performed.

**Results:**

The scores of the RULA and the scores of COPM in the domain of satisfaction and performance during ginger peeling are improved during the post-test and the follow-up period (p < 0.05) The scores of WAI show no statistical significance when comparing the 3 periods (p = 0.079).

**Conclusions:**

The results indicate that the PE program can effectively reduce ergonomic risk factors and enhance the workability among aging workers who work as ginger peelers.

## Highlights

- Ginger peeler aging workers have risk of work-related injury.
- Short period participatory ergonomics program can reduce ergonomic risk factors and enhance work ability.
- The effects of the program remain even during the follow-up period.
- Participatory ergonomics programs can be applied for ginger peeler aging workers in community context.

## INTRODUCTION

At present, the total number of older adults in Thailand, as of 2023, was approx. 12 million people, accounting for 19.40% of the country's total population. This figure evidently shows that Thailand has fully transitioned into an aged society [1]. Currently, the United Nations and many countries use the age ≥65 years to present statistics, data, and indicators related to population age structure. In Thailand, “the elderly” is defined as a person aged ≥60 years as well as the retirement age for the government sector is currently 60 years, while in the private sector, it is 55–60 years [1].

In 2022, regarding the number of older adults who were still in the workforce, there were 4.74 million people, accounting for 36.1% of the country's population of older adults. It was found that older workers worked approx. 39 h/week, averaging 7–8 h/day. Most of them worked in agriculture and fishery, accounting for 57.1% of older workers [1]. There were several reasons why the elderly continued to work, such as the desire to provide income to sustain both themselves and their families' livelihood, to improve their lives, to be proud of themselves, the need for self-esteem recognition, to provide encouragement and motivation to continue living [2]. In this regard, encouraging older adults who want to continue to work actively, according to their potential and readiness, can be used as a means of reducing the impact on countries that are transitioning into an aging society. Such a measure will also improve the mental health of older adults, and allow them to earn enough income to sustain their lives, as well as alleviate the country's labor shortage problem. The policy of productive aging and job placement follows Thailand's National Plan of Action on Older Persons [1]. The retirement age plans to extend to 65 years and promote continuous employment among the working-age population for both the private and government sectors. Enhancing the working capabilities of older people is an important issue. One of the limitations that older workers have to face is the age-related deterioration of bodily functions and their cognitive ability [3]. Older adults will require more time to learn new things. They will be less concentrated, and their associative reasoning and information processing will be noticeably slower as they age. The bodies of older adults are also deteriorating. They will have less muscle strength, limited joint movements, poor gesture control, and decreased body balance. Besides age-related deterioration, older adults normally suffer from chronic diseases, such as diabetes, high blood pressure, heart disease, musculoskeletal disease. As a result, older workers are at more significant risk of injury and work hazards than people of working age, causing older workers to have to take more leaves of absence [4,5]. This will ultimately reduce their overall work efficiency.

In Wat Puranawas Community in Thailand, an older adult club was established with the aim of helping and taking care of each other. The Wat Puranawas Community encourages older adults to continue working so they may help sustain themselves and their families. One of the jobs that the Community continually arranges for its members is to work as a ginger peeling worker for animal feed manufacturers. The work processes are, as follows: representatives of the Community will pick up batches of fresh gingers from the manufacturers and then distribute them to workers in the Community. Older workers are responsible for peeling gingers and cutting them into the desired sizes. The Community's representatives will later pick up the processed gingers from workers' homes and deliver them to the employers. In this regard, a closer look at the postures of older workers reveals certain risks of bone and muscle injuries from work [6,7]. A research study by Kumar et al. [6], who studied the risk factors among pineapple peelers in India, reported that pineapple peelers were at risk of ergonomic injuries because they usually bent their bodies forward and sideway, as well as lowering their necks. Their works also required their wrist to withstand lots of force, with their arms crossed over the midline and wrists turned away from the midline, while they used knives of various sizes to peel the pineapple along the curved surface. More importantly, they maintained the same position and repeated the same movements for many hours.

The participatory ergonomics (PE) program is a term for programs that emphasize the participation of workers in the planning, designing, and improving the characteristics or behaviors of their works while observing the ergonomic principles, with the help of advisors who possess knowledge in ergonomics, so workers will be able to apply knowledge in ergonomics in their works [8–11]. The primary assumption of the PE program is that workers are experts in their line of work. They possess the knowledge and skills that allow them to identify and analyze any work-related problems, in order to develop and implement solutions that will effectively improve productivity and reduce risks of occupational diseases. Previous research studies found that the PE program could be used effectively to prevent musculoskeletal disorders and work-related injuries, as well as to improve the overall performance of workers from various fields [12–16]. Several types of PE programs have been designed for the specific needs and demands of workers of different organizations and fields of work. These PE programs are varied by various components, for example, the level of participation of participants and experts, the duration of the program, the underlying conceptual framework used for identifying and resolving the problems. However, there are merely a handful of PE programs that studied potential risks of work injuries and the effect of PE programs on aging workers who work in communal enterprises. Accordingly, this pilot clinical trial was proposed with the objectives of assessing the effects of a PE program on assessing the risks of work injuries due to working postures and assessing workers' work ability and satisfaction with their work, among aging workers who worked as ginger peelers in the Wat Puranawas Community.

## MATERIAL AND METHODS

### Research design

The present research was designed as quasi-experimental research and was conducted among a single group of the population. This study evaluated the effectiveness of the PE program for reducing ergonomic risks and promoting work ability among aging workers who work as ginger peelers in the Wat Puranawas Community, a community in Thawi Watthana District, Sala Thammasop Sub-district of Bangkok, Thailand. The *Rapid Upper Limb Assessment* (RULA) was used to collect and assess the data regarding the risks of working postures; and used the *Canadian Occupational Performance Measure* (COPM) Thai version, combined with the *Work Ability Index* (WAI) Thai version to assess workers' work ability. The author then compared the results from 3 periods of time include prior to the application of the PE program, 2 weeks during the application of the PE program, and during the follow-up period after the conclusion of the PE program. Ethical approval for the study was obtained from the Institutional Review Board's Ethical Committee (COA No. MU-CIRB 2023/189.2212). The study was performed in accordance with the ethical standards as laid down in the Declaration of Helsinki and its later amendments or comparable ethical standards. All participants signed a consent form before recording the data.

### Participants

Participants were older workers who worked as ginger peelers in the Wat Puranawas Community, during the period of January–June, 2024. There was a total of 16 aging workers during this period. The samples were selected using a purposive sampling method, according to the following inclusion criteria: participants must be older adults, >60 years old, who worked as ginger peelers in the Wat Puranawas Community for at least 3 months. The exclusion criteria were, including, having an MMSE score of <23 pts, having been in an accident during the past 1 year, or still suffering from accidents or being diagnosed with neurological diseases that prevent them from working or participating in the study until the end.

### Instruments

The first instrument was a questionnaire used for gathering general information about participants, including their sexes, ages, history of congenital diseases, work experience, education level, daily work hours, etc. The second instrument was the RULA, Thai version, which was used for assessing the risks of work injuries due to working postures. For this purpose, cameras were used to record workers as they worked different tasks. The RULA measures postural risk factor during the task by using a scoring system [17]. Workers were given a score from 1–7 for their tasks. The result was then categorized into 4 levels, as per the total score given:

– !1–2 pt – level 1: working postures are acceptable but there may be ergonomic problems if such postures are repeated for a prolonged period;
– 3–4 pts – level 2: further study is required, workers' performance should be continually monitored, and it may be necessary to redesign the works;
– 5–6 pts – level 3: working postures are not suitable, further study and improvement of the works should be conducted as soon as possible;
– >6 pts – level 4: the working postures represent ergonomic problems and must be improved immediately.

The COPM, Thai version, was used to assess workers' productivity domain. The COPM is a standardized client-centered outcome measure that measure progress of the setting goal during intervention to measure progress [18,19]. The participants would rate the work processes by their level of significance, giving them a score of 0–10 pts (a score of 0 pts means non-significant, and a score of 10 pts means extremely significant.) Afterward, the participants were asked to choose important processes, using the previously assigned scores of significances, in order to identify the participants' performance with such processes, by giving them a score of 0–10 pts (a score of 0 pts means participants were unable to perform the process and a score of 10 pts means participants were extremely skillful with the process.) The participants were then asked to rate the level of their satisfaction with different processes, by giving them a score of 0–10 pts (a score of 0 pts means participants were very unsatisfied while 10 pts means participants were very satisfied).

The *Work Ability Index* (WAI), Thai version, is a self-assessment questionnaire that workers can use to assess their work ability [20,21]. The questionnaire contains 7 items that gather data about workers' work ability, namely, their current work ability, workers' abilities and nature of their work, any illnesses and diseases diagnosed by doctors, injuries that prevent them from working, leaves of absence they took due to health problems, the expectation of future work, and assessment of mental health at work. The questionnaire yields a total score of 49 pts which can be categorized into 4 levels: low (7–27 pts), medium (28–36 pts), good (37–43 pts), and excellent (44–49 pts).

### Procedure

Following the ethics approval, the author (primary investigator) contacted the leader of the Wat Puranawas Community to request his permission to conduct research and implement the PE program in the Community, as well as to invite members of the Community to participate in this research project. The author contacted interested members to make an appointment. Prior to data collection, the author delivered a letter to participants. This letter explains the purposes of the present study before participants performed their consent.

A research assistant, who is an occupational therapist and does not know the objectives of the present research, was trained in the use of research instruments and assigned the responsibility of collecting the data during a period of 3 days prior to the day that the author initiated the PE program with participants. The data collection was conducted at participants' workplaces, namely, at the Community Center or participants' homes. Participants were interviewed by the research assistant, using the WAI and the COPM. Afterward, the author assessed the ergonomic risks using the RULA while participants performed their work. The work of ginger peelers can be divided into 2 main steps: peeling and cutting. Participants were asked to perform their work while being recorded by a video camera, for a period of 15 min. Later, the author conducted the PE program with participants twice. Each session took 60 min to complete. Data were collected twice thereafter, 2 weeks and 4 weeks after the conclusion of the PE program. All data collections were conducted at the same workplace of each participant.

### Interventions

Participants attended the PE program twice and each session lasted 60 min. During the first session, participants studied and assessed the risks of injury from working postures, using the data that the author previously collected and analyzed. Participants identified additional problems and risk factors of the 2 steps of ginger peeling. At the end of the session, the author discussed with participants, asking them to provide their opinions and to share their experiences, in order to determine the possible improvement of the work processes and to create a guideline for proper operation. The proposed improvement aimed at improving working postures, enhancing work safety, adjusting the duration of work, and improving the equipment and the working environment. For this purpose, the researcher served as a coordinator to implement these improvements while educating participants on the knowledge about ergonomics, if participants asked. The second session was for the implementation of the guideline for improvement, whereas participants were asked to study the guideline for proper operation that the author prepared from the data collected during the first session. Participants were then asked to perform their tasks while observing the proper working postures, in a working environment that has been improved. Their works were recorded by a video camera. The video data was later shown to participants so they could observe their working postures. Afterward, the author discussed with participants about the result, asking participants to provide feedback on the perceived advantages of the improvement. The researcher then appointed a leader and a team to monitor the ergonomic risks, giving them the task of ensuring that participants will continue to observe the guidelines in the long term.

### Statistical analysis

The statistical analysis used the SPSS v. 18 and used descriptive statistics to describe the characteristics of participants, with normal distribution. After performing the Kolmogorov-Smirnov test, the author that the data were not distributed normally and therefore applied the Friedman test to compare the differences in the results of scores from the RULA, COPM, and WAI assessment forms, using the data collected from 3 periods, namely, the pre-test data, the post-test (2 weeks after the program), and the follow-up (4 weeks after the program). The *post hoc* test used the Wilcoxon test to determine the difference between these periods. The level of statistical significance was p < 0.05.

## RESULTS

There was a total of 14 participants who participated of the research project. All participants attended both sessions of the PE program and attended the 3 periods of assessments. The socio-demographic information of participants is shown in Table 1.

**Table 1:** Socio-demographic information of participants – the aging workers on ginger peelers at Wat Puranawas community, Thailand, January–June 2024

Variable	Participant (N = 14) [n (%)]	M±SD
Sex		
male	3 (21.43)	
female	11 (78.57)	
Age [years]		69.30±6.25
Medical condition		
musculoskeletal injuries	8 (57.14)	
diabetes	6 (42.86)	
hypertension	6 (42.86)	
hypercholesterolemia	6 (42.86)	
thyroid diseases	1 (7.14)	
heart diseases	1 (7.14)	
Education level		
uneducated	4 (28.57)	
elementary education	8 (57.14)	
secondary education	2 (14.29)	
Work experience as a ginger peeler [years]		6.80±3.24
Daily work time		
5–6 h	3 (21.43)	
7–8 h	6 (42.86)	
9–10 h	4 (28.57)	
11–12 h	1 (7.14)	
*Canadian Occupational Performance *Measure* (COPM) score*		
ginger peeling		8.7±1.83
ginger cutting		8.3±1.95

**Figure 1:**
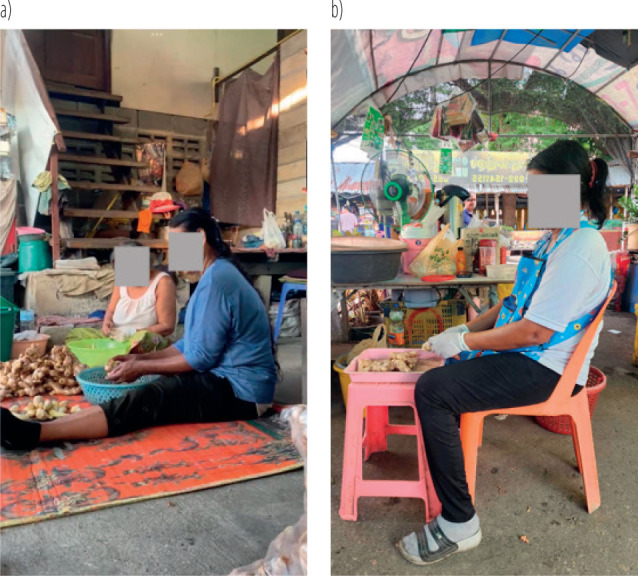
Adjust the working posture and working station according to sit upright with a backrest (the upper arm is neutral position while the forearm and wrist work below the elbow joint): a) pre-participatory ergonomic program, b) post-participatory ergonomic program among the aging workers on ginger peelers at Wat Puranawas community, Thailand, January–June 2024

**Figure 2:**
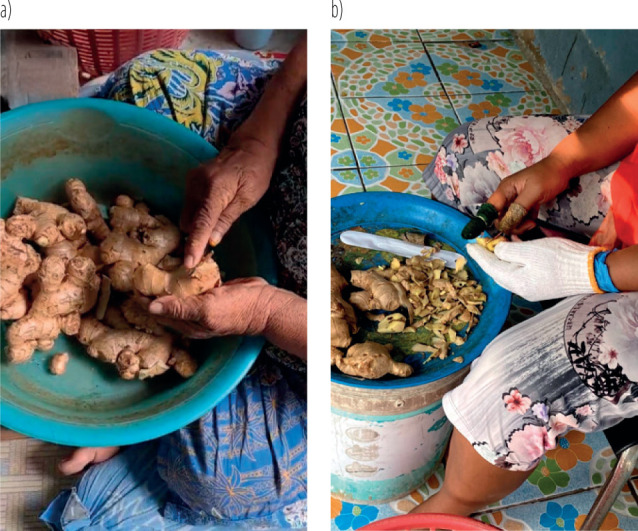
Use of appropriate knife, gloves and proper handing technique during ginger peeling: a) pre-participatory ergonomic program, b) post-participatory ergonomic program among the aging workers on ginger peelers at Wat Puranawas community, Thailand, January–June 2024

**Figure 3:**
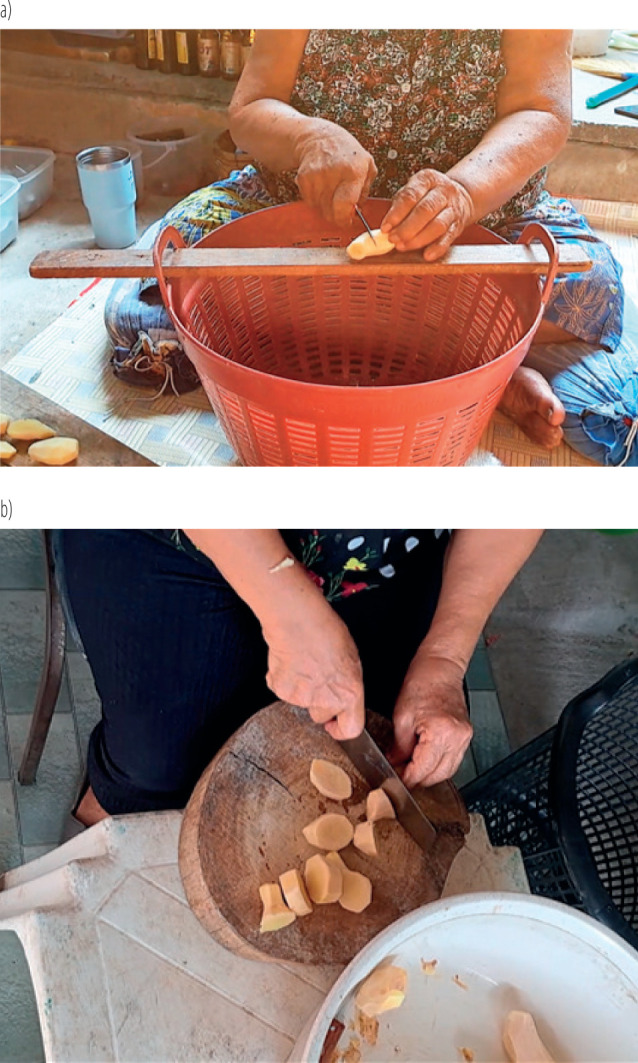
Changing the cutting board, knife, and adjust postural alignment of wrist during ginger cutting: a) pre-participatory ergonomic program, b) post-participatory ergonomic program among the aging workers on ginger peelers at Wat Puranawas community, Thailand, January–June 2024

While participants attended the PE program, the researcher discussed with participants and summarized risk factors of work injuries and solutions, in terms of improvement of the working postures, enhancing the work safety, adjusting the work hours, improving the equipment, and improving the working environment. The details are summarized and presented in Table 2 below. Several examples of the interventions implemented in this study are displayed in Figure 1–3.

**Table 2:** The risk factors of work injuries and solutions among the aging workers on ginger peelers at Wat Puranawas community, Thailand, January–June 2024

Risk	Participants [n (%)]	Solution
Abnormal sitting postures	14 (100)	– adjust the working postures – adjust the workstation – self-monitor – use ergonomic chairs
Repetitive movements	13 (92.85)	– stretching – muscular relaxation techniques – schedule resting session – self-monitor
Excessively exert force	12 (85.71)	– use the stronger muscle – check the sharpness of the knife – adjust the handling posture – use the assistive machine
Prolong working period	11 (64.70)	– adjust the duration of work – schedule resting session
Injuries from knife cuts	4 (28.57)	– adjust the handling posture – adjust the knife – wear gloves
Exposure to ginger's chemicals	5 (36.71)	– education of the chemical hazard – wear gloves – hand washing and first aids
Heat exhaustion	6 (42.85)	– temperature and ventilation control – prevent dehydration

Photo: Surachart Thongchoomsin

Photo: Surachart Thongchoomsin

Photo: Surachart Thongchoomsin

### The results of the PE program

The Friedman analysis reflected significant intra-group changes in the score of RULA, both in the ginger peeking step (χ^2^= 13.273, p = 0.001) and the ginger cutting step (χ^2^= 12.563, p = 0.002). At post-program, significant improvements were found in both the ginger peeling step (p = 0.006) and the ginger cutting step (p = 0.008). The scores of RULA also showed statistical significance when comparing the scores of the pre-program period and the post-program period (the ginger peeling step p = 0.006 and ginger cutting step p = 0.008); but did not show any statistical significance when comparing the scores of the post-program period and the follow-up period (the ginger peeling step p = 0.317 and ginger cutting step p = 0.414).

The scores from the COPM, also showed significant changes from the baseline values in both performance scores (the ginger peeling step χ^2^= 12.000, p = 0.002 and the ginger cutting step χ^2^ = 16.00, p = 0.000) Significant changes also found in the satisfaction scores (the ginger peeling step χ^2^ = 8.824, p = 0.012 and the ginger cutting step χ^2^ = 15.077, p = 0.0010) However, the scores from WAI were not significantly different between the pre-program, post-program, and the follow-up periods (χ^2^ = 5.067, p = 0.079) (Table 3).

**Table 3:** Comparison of the *Rapid Upper Limb Assessment* (RULA) scores, the *Canadian Occupational Performance Measure* (COPM) score in satisfaction and performance and *Work Ability Index* (WAI) score after attending the participatory ergonomic program among the aging workers on ginger peelers at Wat Puranawas community, Thailand, January 2024 – June 2024

Variable	Outcome (M±SD)			χ^2^	p	p		
	pre-program	post-program	follow-up			pre-program–post-program	pre-program–follow-up	post-program–follow-up
RULA								
ginger peeling	5.60±0.70	4.20±0.42	4.00±0.67	13.27	0.001**	0.006**	0.010**	0.317
ginger cutting	5.80±0.63	4.40±0.84	4.60±1.17	12.56	0.002**	0.023**	0.010**	0.194
COPM								
ginger peeling								
satisfaction	8.80±1.32	9.30±0.82	9.80±0.42	8.82	0.012**	0.025**	0.039*	0.79
performance	8.50±0.85	9.30±0.82	9.70±0.48	12.00	0.002**	0.023**	0.010**	0.194
ginger cutting								
satisfaction	8.40±1.17	8.80±1.40	9.80±0.42	15.07	0.001**	0.009**	0.010**	0.180
performance	8.70±0.82	9.80±0.42	9.80±0.42	16.00	0.000**	0.009**	0.032*	0.092
WAI	41.85±4.00	44.80±2.86	44.80±2.86	5.06	0.079	0.054	0.067	0.833

Follow-up – 4 weeks after the program; post-program – 2 weeks after the program.

* p < 0.05;

** p < 0.001.

## DISCUSSION

The main aim of this study was to evaluate the effectiveness of a PE program, focusing on decreasing ergonomic risk factors and increasing the work ability of aging workers who worked as ginger peelers. The results revealed that older workers faced significant ergonomic risks, whereas according to the analysis result of their working postures, using the RULA, the average score of the ginger peeling step was 5.6 pts and the average score of the ginger cutting step was 5.8 pts, resulting in the risk level 3, that is, these older workers faced moderate risks, their work processes should be reviewed, and their working postures should be improved. In addition, as the participants to identify and analyze the problems, as part of the PE program, different risk factors have been found, for example, awkward posture, repetitive tasks with excessive exertion of force, prolonged work period, insufficient rest and brake, the use of sharp equipment, and inappropriate working environment, as shown in Table 2. These findings are in line with the findings of a research study by Kumar et al [6], which reported that 89.4% of pineapple processing workers who engaged in the step of peeling reported RULA scores of levels 3–5, resulting in a high prevalence of pain in their shoulders (41.1%), upper arms (37.1%), and lower back (45.7%). Another research study by Indratula et al. [22], which studied work behaviors and work-related injury among longan fruit peeling workers, revealed several risk factors, such as maintaining the same postures for a prolonged period (96.30%), the workplace being hot and stuffy (82.20%), and working with sharp tools (91.10%). These risks led to a higher proportion of work-related injuries, namely, hand and wrist pain (64.40%) and leg and knee pain (56.30%).

The present study solved work-related problems using the PE program to analyze the tasks, adjust the working postures and work processes, and improve the working environment and equipment appropriately. Though only 2 sessions of the PE program were implemented, the result of the post-program period showed significant changes in the RULA scores, as well as in the performance and satisfaction scores of COPM. In addition, the result of the PE program continued to show even during the follow-up period. The PE program relies on workers' participation, allowing workers to identify the risk factors so experts can analyze such risk factors and determine the solutions, create the guidelines for proper operations, and implement such guidelines to improve operations [8,10]. Lin et al. [15] studied the effectiveness of the PE program in reducing the working posture-related risks among dentists by using the RULA, as well as using the WAI to analyze work ability.

The PE program encourages participants to participate in the efforts to make improvements, as well as to change their work behaviors. The program allows participants to share their work experience and opinions with their peers, in order to determine the solution for improvement, under the supervision of a team leader who provides feedback to the experts. Therefore, the PE program is very suitable for older adults and the program's result continues to exist for a long time even though the program takes merely several short sessions to implement. The result is in line with a research study by Burgess et al. [13] who implemented the PE program with coal miners. They found that allowing workers to participate in the solution-finding effort resulted in a greater sense of belonging. Participants were more motivated and determined to improve. They were also able to observe the guidelines for impro vement more thoroughly and to solve the problem more efficiently. Most importantly, the PE program produced a high level of motivation for change, in comparison to simply attending training courses arranged by the researchers. On the other hand, a research study by Rasmussen et al. [16] studied the result of implementing the PE program among 594 eldercare workers, who attended 2 sessions of the PE program and 1 follow-up session, each session lasting 1h. The result showed that the PE program had significantly reduced physical work demand and the incidence of back pain.

For the present study, the WAI scores showed no significant change after the implementation of the PE program. The WAI assessment is related to the work capacity [20]. The work capacity is based on the balance between a person's resources and work demands. The basis for ability is health and functional capacity, work capacity is also determined by professional knowledge and competence, values, attitudes, and motivation to work [23]. The objectives of the PE program designed for the present study were improving the working postures, working environment, and equipment. The program does not focus on managing the underlying health condition and age-related deterioration of older adults, whereas chronic health condition, such as diabetes, hypercholesterolemia, hypertension, and thyroid diseases were highly prevalent among participants, all of which greatly affect workers' work ability [24,25]. Van den Berg et al [26] are also reported that poor musculoskeletal capacity, older age, obesity, poor physical work environment, and high physical workload were the factors associated with poor work ability index. Therefore, adjusting the PE program to also help solve and alleviate the health condition of workers will be greatly beneficial to older workers. In this regard, Early et al. [25] implemented the principles of activity analysis, modification of job demands, health promotion, and successful aging to encourage older adults to stay in the workforce. In addition, the time interval between before and after the modification of the jobs was too short (2 weeks after the program) to allow for changes in the WAI level. Increasing the number of training sessions and extending the re-assessment period should reveal more changes in work capacity.

### Limitations

There are some limitations because the present study was only conducted in 1 community and the task of ginger peeling is not at all complicated. Moreover, other than ginger peelers the present study did not include employees from other lines of work. In general, a PE program should use a multi-disciplinary approach and involve employees of different roles, including executives and employees, in the development and implementation of solutions. Therefore, due to the nature of the PE program designed for the present study, it may not be appropriate to generalize the result to other fields of work or industries, especially those that involve far more complicated work processes and many workers who engage in different tasks. Another limitation of the present study was that the PE program used herein only included a follow-up period of 1 month. Increasing the duration of the follow-up period will allow researchers to learn the result of the program in the long term.

## CONCLUSIONS

The present study provides evidence on the effectiveness of the PE program on aging workers on ginger peeler group and suggests that the program is beneficial to their job performance and helps prevent work injury. This PE program aims to improve working postures, enhance work safety, adjust the duration of work, and improve the equipment and the working environment. This PE program can be more effective when it takes into account the specific needs of this population. The studies of older workers in different lines of work are now needed, in order to examine the potential benefits of PE programs that aim at improving workers' performance and preventing work-related injuries, especially among older workers who work in communal enterprises.
